# Socio-ecological factors determine crop performance in agricultural systems

**DOI:** 10.1038/s41598-020-60927-1

**Published:** 2020-03-06

**Authors:** Libère Nkurunziza, Christine A. Watson, Ingrid Öborn, Henrik G. Smith, Göran Bergkvist, Jan Bengtsson

**Affiliations:** 1 Department of Crop Production Ecology, Swedish University of Agricultural Sciences (SLU), P O Box 7043, SE 75007 Uppsala, Sweden; 2 Scotland’s Rural College (SRUC), Craibstone Estate, Aberdeen, AB21 9YA Scotland; 3 Centre for Environmental and Climate Research & Department of Biology, Lund University, SE 223 62 Lund, Sweden; 4 Department of Ecology, SLU, P O Box 7044, SE 75007 Uppsala, Sweden

**Keywords:** Plant sciences, Ecology

## Abstract

Agricultural production systems are affected by complex interactions between social and ecological factors, which are often hard to integrate in a common analytical framework. We evaluated differences in crop production among farms by integrating components of several related research disciplines in a single socio-ecological analysis. Specifically, we evaluated spring barley (*Hordeum vulgare*, L.) performance on 34 farms (organic and conventional) in two agro-ecological zones to unravel the importance of ecological, crop and management factors in the performance of a standard crop. We used Projections to Latent Structures (PLS), a simple but robust analytical tool widely utilized in research disciplines dealing with complex systems (e.g. social sciences and chemometrics), but infrequently in agricultural sciences. We show that barley performance on organic farms was affected by previous management, landscape structure, and soil quality, in contrast to conventional farms where external inputs were the main factors affecting biomass and grain yield. This indicates that more complex management strategies are required in organic than in conventional farming systems. We conclude that the PLS method combining socio-ecological and biophysical factors provides improved understanding of the various interacting factors determining crop performance and can help identify where improvements in the agricultural system are most likely to be effective.

## Introduction

Crop yields are influenced by climate, soil type, and numerous decisions that farmers make each year regarding fertilizer use, weed and pest management, crop and varietal choice, tillage and many other factors. It is also widely accepted that both environmental factors outside the control of farmers and landscape characteristics can influence yields^1–4^. While it is well known that previous cropping strategies and land management history can influence yield via “rotation” and “memory” effects^5,6^, it is difficult to quantify the impact of previous management on crop yield due to the complex interactions between management decisions and the biophysical environment. Unravelling these relationships requires a methodology that can examine relationships between different types of socio-ecological and biophysical variables.

Factorial experiments currently dominate crop performance evaluation in crop sciences. However, it has been argued that they do not provide comprehensive and holistic understanding of cropping systems, as they are unable to account for more than two or three factors^7,8^. In reality a multitude of factors affect crop performance simultaneously and often with trade-offs that are difficult to evaluate. Factorial experiments are commonly used to evaluate the yield potential of given genotypes under different agro-ecological conditions^9,10^ or technologies, but deliberately keeping most environmental factors constant or explicitly manipulating only one or a few environmental factors. The predominance of crop performance evaluation based on field experiments under relatively controlled conditions has led to the dominance of univariate and bivariate methods in agricultural research, and ignores the nonlinear effects, feedbacks and interactions observed in many ecological and social-ecological systems^11^. In addition to the high costs associated with experiments where the approach is to keep as many variables as possible under control, the large difference between yields obtained in experimental studies and on-farm yields demonstrates the discrepancies between expected and real farm outcomes, which is particularly the case for less intensive farming systems^12,13^. This calls for alternative assessment methods that qualitatively and quantitatively integrate disparate causes of variation in crop performance, such as the temporal and spatial boundaries used in the evaluations. These boundaries are dependent on farmer decisions, which are often guided by socio-economic conditions including finances, technologies and experience.

During recent decades, process-based models integrating genotype, management and environment have been developed to aid tactical and strategic decision-making^14,15^. However, although these methods often provide insights at the system level where many factors are integrated in the model, process-based modelling suffers from large uncertainties^16,17^. Simulated crop performance obtained with mathematical functions often deviates greatly from the observed data^16^. The mathematical formulation of the functional relationships between variables requires a deep understanding of the system under study, parameters estimated with high precision and model-specific data for calibration and validation that are often scarce.

The integration of biophysical and socio-economic models has mainly been carried out at larger scales than that of fields^18–21^ and the results therefore cannot be used to explain crop performance. Hence, there is a need to develop methods that facilitate understanding of crop performance at the field level as a result of different biophysical and socio-ecological conditions. The development of such multidisciplinary approach would constitute a step forward, particularly if multiple independent variables and multiple responses could be included in the analyses. Projections to latent structures (PLS) can integrate multiple factors from a range of disciplines in a single analysis, thus enabling the inclusion of socio-economic and management variables in the same analytical framework as biophysical variables^22–24^.

Organic and conventional farming systems generally tackle problems related to crop production in different ways. While conventional farmers often rely on targeted short-term solutions, such as the application of agrochemicals, many organic farmers take a strategically different approach utilising longer-term solutions (preventive and proactive rather than reactive) at the systems level^25,26^. This leads us to hypothesize that historical farm and field management practices and spatial location of fields in the landscape have a greater influence on crop performance on organic than conventional farms. In this study, we aimed to identify whether different factors influence crop performance on organic and conventional farms using data from surveys on long-term management practices, soil variables and spring barley (*Hordeum vulgare*, L.) performance indicators including 34 farms situated in two agro-ecological zones in Sweden (see Table 1 in Material and Methods section for all included variables). We also quantify the variation in crop performance among farms with respect to the time since transition to organic farming. Finally, we examine how well individual crop performance indicators can be explained by the PLS analysis in order to suggest recommendations of relevant factors to include and vary in future field studies and experimental designs, thus improving cost effectiveness of such studies. Using this approach, we explore the usefulness of PLS for expanding the temporal and spatial boundaries of the evaluation of agro-ecosystem and helping to disentangle the causes of variation of crop performance in different agricultural systems.

**Table 1:** Farm types (FT), year of conversion to organic farming (YCOF) and barley cultivar and type in 2012.

Uppsala County agro-ecological zone					Scania County agro-ecological zone				
Farm ID	FT	YCOF	Cultivar	Barley type	Farm ID	FT	YCOF	Cultivar	Barley type
U1	OOF	2002	Baronesse	feed	S1	OOF	1996	Justina	feed
U2	CF	—	Tipple	malting	S2	CF	—	Tam Tam	malting/feed
U3	CF	—	Tam tam	malting/feed	S3	OOF	1995	Justina	feed
U4	CF	—	Tipple	malting	S4	OOF	1999	Justina	feed
U5	CF	—	Columbus	malting	S5	YOF	2010	Mercada	feeder
U6	CF	—	Tipple	malting	S6	CF	—	Tam Tam	malting/feed
U7	OOF	1996	Mitja	feed	S7	OOF	2001	Orthega	feed
U8	OOF	1994	Mitja	feed	S8	CF	2008	Luhkas	feed
U9	OOF	1989	Columbus	malting	S9	CF	—	Quench	malting
U10	YOF	2009	Mitja	feed	S10	YOF	2009	Anakin	feed
U11	OOF	1987	Baronesse	feed	S11	OOF	1999	Justina	feed
U12	YOF	2009	Mitja	feed	S12	CF	—	Tipple	malting
U13	YOF	2012	Mercada	feed	S13	CF	—	Tipple	malting
U14	OOF	1996	Gengel	Feed	S14	CF	—	Anakin	feed
U15	CF	—	Tipple	malting	S15	YOF	2012	Luhkas	feed
U16	YOF	2007	Orthega	feed	S16	YOF	2010	Justina	feed
U17	YOF	2008	Otira	feed	S17	YOF	2012	Luhkas	feed

## Material and Methods

### PLS description

#### Usefulness of PLS

Projections to latent structures (PLS), a more correct term for Partial Least Squares^27^, is a powerful multivariate method that is able to integrate data from different scientific disciplines in a single analysis^22,24^. It has minimum demands in terms of sample size, residual distribution and measurement scales^28^, while at the same time being able to handle a large amount of information on a relatively small number of independent observations. Allowing an analysis of crop performance that includes the many noisy and collinear variables related to farmers’ management choices as well as ecological, soil and landscape variables, the method is able to recognize farming systems and agro-ecosystems as complex social-ecological systems rather than simple bio-physical systems. Agro-ecosystems encompass ecological and decision networks and management inputs that are connected to one another and perform different functions leading to the provision of a wide range of ecosystem services, including crop yield and quality^29^.

#### Technical description

PLS is a partial least squares regression analysis and is used to find the relationships between two matrices *X* and *Y*^22^. It is a latent variable approach to modelling the covariance structures in these two spaces. While PLS is fairly well known in social sciences, marketing, psychology and education, it is also used for example in chemometrics^24,27,30^. One of the reasons for using PLS is the costs associated with including a large number of objects (individuals) in classical analyses. As an extension of Principal Component Analysis (PCA), PLS derives its usefulness from its ability to analyse data with many, noisy, collinear, and even incomplete variables in both X and Y. Details on the origins, evolutions and applications of PLS in the social sciences have been published previously, as well as the presentation of related methods in the family of multi-block analysis^28,30,31^. The performance of the PLS regression models improves with relevant X-variables that explain the most variation in Y variables. The PLS model diagnostic of its appropriateness, i.e. a model with optimal balance between fit and predictive ability^32^, is based on parameters R^2^Y (explained variation) and Q^2^Y (predictive ability). Details on the significance and evaluation of the goodness of fit (R^2^Y) and goodness of prediction (Q^2^Y) through the cross validation (CV) method under PLS have also been explained in a previous study and in other publications^22–24^. The goodness of fit and prediction of each response variable are obtained with PLS coefficients and the root mean square error (RMSE, %) is calculated to assess the predictive ability.

##### Farm choice and description

Thirty four farms were selected in two agro-ecological zones; the Swedish central eastern county (Uppsala County, around 60°N, 18°E) and the Swedish southern county (Scania County, around 55°N, 13°E). Due to latitude differences the two agro-ecological zones, hereafter called regions, differ in terms of climate that affects the growth and development of barley and soil processes. For both regions, seventeen farms growing spring barley (the most common annual crop) were selected including conventional farms and organic farms. Within organic farms, time since conversion to organic farming varied from 1 to 26 years, which enabled us to include a broad range of management practices as a result of management skills and experience developed over time. Three groups of farms were considered: conventional farms (CF), young organic farms (YOF) with less than 6 years since transition from conventional farming practices, and old organic farms (OOF) with 11 to 26 years since transition. Farms included mixed arable and livestock systems with cattle, pigs and/or horses in addition to pure arable farms. Sizes of the farms varied from 34 to 700 ha in Uppsala County and from 11 to 260 ha in Scania. The farms were selected to represent the length of the landscape complexity gradient in the regions. The distribution along the gradient went from complex landscapes with many non-crop habitats and forested areas to more homogenous agricultural landscapes with mainly arable land. Care was taken to select farms in such a way that all categories of farms (CF, YOF, OOF) were represented along the whole landscape gradient in each region^33,34^. The selected organic farms were certified by KRAV, the most common Swedish Trademark for organic products.

##### Survey of farm management practices

A questionnaire survey was conducted with the farmers in late 2011 and 2012 to obtain data on management practices on a given barley field for each farm in the present and recent past. Questions were directed to understanding the management at the whole farm level, with special focus on the management practices during the period 2009–2012 on one field per farm where barley was grown in 2012. Farm types, year of conversion to organic farming, and cultivar grown in 2012 were recorded (Table 1). All interviews were conducted on farm. The questions are provided in Table S1, along with the type of answers and corresponding management practices, which were considered for the analysis.

Due to the diversity of possible answers about management practices and resources used on farm, we aggregated them under a set of synthetic variables to reduce the number of independent variables in the analysis. In this way, we reduced the number of possible answers (variables) in the analysis from 132 to 29 variables, out of which 11 related to farm characteristics and 18 related to field management (Table 2). Aggregation procedures were detailed, for example for livestock density index, frequency of organic fertiliser application, etc., in a previous study^23^.

**Table 2:** The 34 explanatory variables used in the projection to latent structures (PLS): 1–3: Farm level description, 4–11: management practices (MP) at the farm level, 12–29: MPs at the field level, 30–34: field level soil parameters.

Farm level description and MP; Symbol (Unit)		Range (Uppsala; Scania)	Variable explanation
1. Time since transition	TST (year)	0–26; 0–18	
2. Farm size	Size (ha)	34–700; 11–260	
3. Landscape heterogeneity index 1 km radius	LHI (−)	−1.4–2.0; −2.1–2.3	LHI^a^ = sin45 × (standardized proportion of semi-natural grasslands+ standardized proportion of field border)
4. Proportion of rotational leys	Leys (%)	0–64; 0–87	Farm area including pasture and permanent pasture
5. Proportion of cereal crops	Grains (%)	18–95; 6–85	Farm area including pasture and permanent pasture
6. Proportion of other crops	Ocrops (%)	0–35; 0–56	Farm area including pasture and permanent pasture
7. Presence of pasture	PP (−)		Dummy variable: present (1) or absent (0)
8. Area with organic fertilizers	OFert-area (ha)	0–380; 5–260	
9. Amount of organic fertilizers	AOFert (ton ha^−1^)	0–30; 0–70	
10. Livestock density index	LDI (−)	0–1.5; 0–3.3	A measure of livestock per hectare of utilized agricultural area including pasture and permanent pasture
11. Straw and residue management	SRM (−)	1–3	Scale from 1–3: where the highest value 3 = always incorporated, 2 = sometimes incorporated and 1 = removed from the farm
**Field level MP (2009**–**2011); Symbol (Unit)**		**Range**	**Variable explanation**
12. Frequency of organic fertilizer (OFe)	Freq-OFe (−)	0/1	0–1: Number of organic fertilizer applications over the 3 years divided by 3
13. OFe application technique	OFe-AT (−)	1/2	Scale 1–2: where 2 = Broadcasting and mulched, 1 = either broadcasting or mulched and 0 = none of the two
14. Mineral N on average	Min-N (kg ha^−1^)	0–175; 0–102	Average of N application over the 3 years
15. Mineral PK applied	Min-PK (−)	0/1	Dummy variable: used (1) or not used (0)
16. Pesticide application	PEST (−)	0/1	Dummy variable: used (1) or not used (0)
17. Straw and residue management	STR-M (−)	0–2	Scale 0–2: where 2 = incorporated and mulched, 1 = either incorporated or mulching and 0 = none of the two
**Field level MP in 2012 Symbol (Unit)**		**Range Variable explanation**	
18. Nitrogen amount from organic fertilizers	Org-N12 (kg ha^−1^)	0–150; 13–167	
19. OFe application technique	OFe-AT12(−)	0–2	Scale 1–2: where 2 = Broadcasting and mulched, 1 = either broadcasting or mulching and 0 = none of the two
20. Mineral N application	Min-N12 (kg ha^−1^)	0–175; 0–103	
21. Straw & residues left on the field before sowing in 2012	SMR-L12 (−)	0/1	Dummy variable: left (1) and removed (0)
22. Sowing date^b^	StdSd (DOY)	121–145; 84–122	Day of the year
23. Seed rate sown	Seed (# m^−2^)	180–220; 100–125	
24. Pea as a preceding crop to barley	PC-pea (−)	0/1	Dummy variable: pea (1) or other (0)
25. Leys as preceding crop to barley	PC-leys (−)	0/1	Dummy variable: leys (1) or other (0)
26. Cereals as preceding crop to barley	PC-cereal (−)	0/1	Dummy variable: cereals (1) or other (0)
27. Use of pesticide	PEST-12 (−)	0/1	Dummy variable: used (1) or not used (0)
28. Barley undersown with grass/clover	US-12 (−)	0/1	Dummy variable: undersown (1) or not (0) of barley
29. Percentage weed cover^c^	Weed (%)	0–33; 0–31	Average of the percentage weed cover of 3 assessments
**Field soil parameters; Symbol (Unit)**			**Variable explanation**
30. Soil mineral nitrogen before fertilisation	SMN1 (kg ha^−1^)	12–57; 16–36	
31. pH	pH (−)	5.6–8.0; 5.7–7.4	
32. Total soil carbon	Tot-C (%)	1.4–12.0; 1.1–3.5	
33. Total soil nitrogen	Tot-N (%)	0.1–0.1.0; 0.1–0.3	
34. Soil clay content^b^	Clay (%)	17–66; 4–35	

Variable abbreviations (with unit given in parentheses), ranges of each region (Uppsala and Scania Counties) and explanation. Dummy variables (0/1), frequencies and indices were dimensionless (−) and no ranges are shown.

^a^The LHI index is based on the proportions of semi-natural grassland and field border in the surroundings of the field (see text for references).

^b^Sowing sates and soil clay content were standardized, to exclude the differences between the two regions, by taking the median of each region as zero.

^c^Indicator of the efficiency of weed control.

##### Barley performance indicators and weed cover

On each farm, one spring barley field was selected as a standard study crop, which is the second most important cereal in Europe^35^, for both humans and livestock. In Sweden, barley and winter wheat are the main cereal crops in terms of cultivated area with around 318 and 476 kha, respectively, in 2018^36^. In 2017, the barley production was estimated at 447,900 tonnes in Scania and 145,800 tonnes in Uppsala county^36^. Spring barley was chosen as a model crop for its importance in terms of production but also because it is better distributed among different farm types; arable farms, mixed farms and specialised livestock farms. For each field, the landscape complexity around the field was determined according to the definition of landscape heterogeneity index^33,34^. In the case of more than one barley field on a given farm, a high landscape index (in the radius of 1 km) was the main criteria for choosing which barley field to study in order to increase the landscape complexity gradient when examining diversified management practices between conventional and organic farms. The LHI index is based on the proportions of semi-natural grassland and field border in the surroundings of the field^23,37^.

In 2012, seven barley performance indicators (BPIs) were measured in the selected spring barley field on each farm (see above). The BPIs included *N concentration* in the biomass (grain and whole biomass), and *dry matter* (DM) *production* at two growing stages: BBCH 31(stem elongation) and BBCH 87 (ripening: hard dough) according to Lancashire^38^. Biomass samples (4 random quadrats of 0.25 m^2^ per field, in total 1 m^2^) were cut at 5 cm above the ground and oven-dried at 60 °C for at least 24 hours. At harvest, BBCH 87, DM of straw and grain were separated. Samples were taken at a minimum of 20 meters from the edge of the field. Percentage weed cover was visually estimated during barley growth and an average percentage weed cover estimated on 18, 25 July and 2 August 2012 (for which data were complete for all the fields) was included as a variable affecting the BPIs beside the management practices. At the harvest, BBCH 87, the number of ears per sample was counted. Nitrogen concentration in the straw and grains was determined with an elemental LECO 2000CN analyzer.

##### Soil characteristics measurements

On each selected barley field, soil mineral N (SMN) was measured in on samples collected before fertilizer application early in the 2012 growing season (Table 2). In addition, total soil C and N (LECO 2000CN analyser), soil pH (1:2.5 H_2_O) and texture were measured in each selected barley field. The percentage of clay was used to represent the variation in soil texture.

##### PLS application

**Figure 1:**
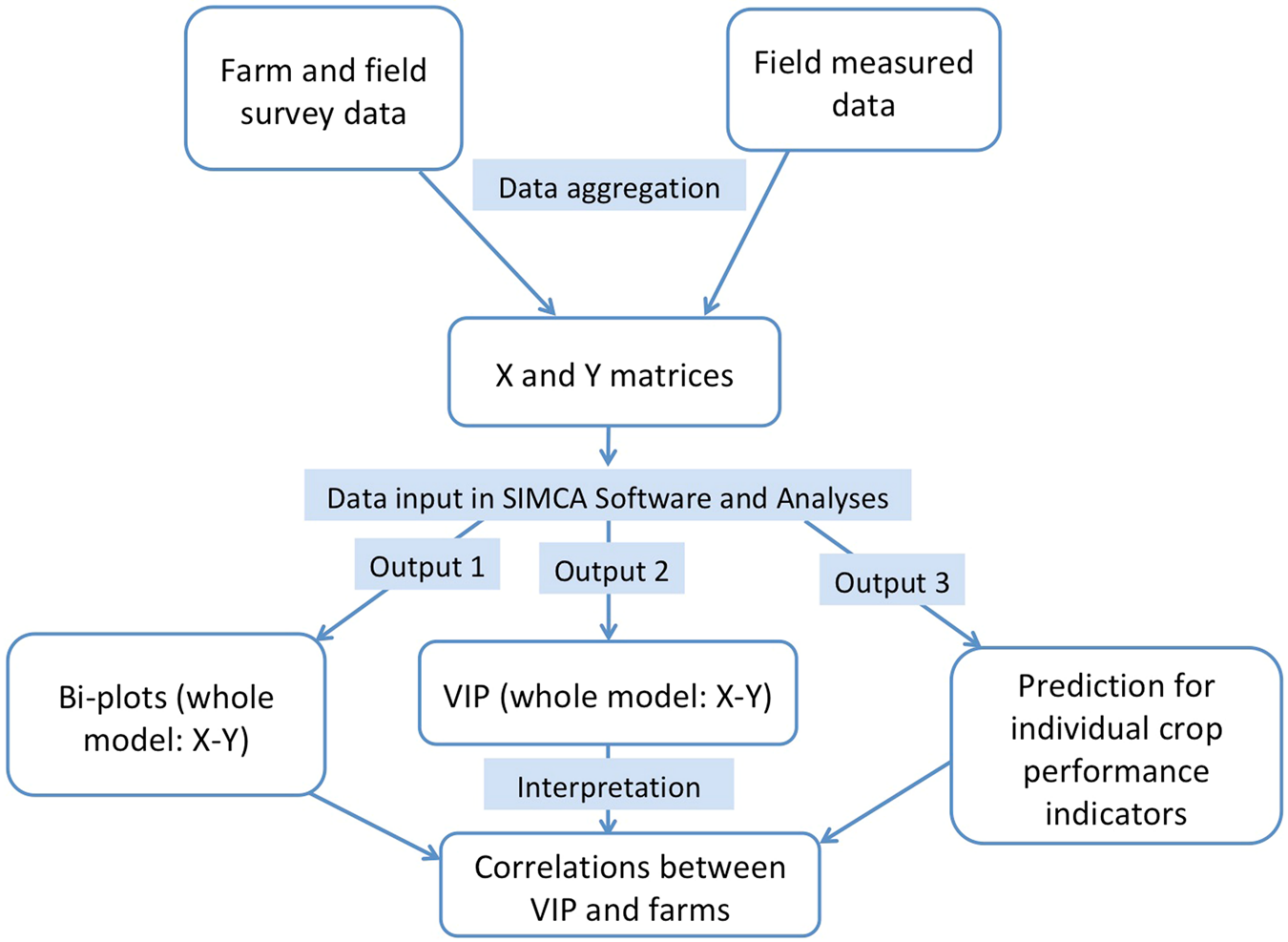
Schematic PLS method for on-farm data analysis linking socio-ecological factors and crop performance indicators. The analysis follows many steps and several combinations of variables to find the best model.

In this study, PLS was used to examine how different sets of explanatory variables (X) were related to the set of barley performance variables (Y) (see a schematic method description in Fig. 1). The X consisted of 28 management practices (aggregated from 100 variables originally measured), 5 soil and 1 landscape characteristics (X-matrix, 34 variables) and Y was barley performance indicators (Y-matrix, 7 variables). Variables influenced by regional location, e.g. because of climate differences, were standardized (e.g., sowing date, soil clay content). Each farm was considered as an object, a unit with complex interactions in the system. For farm level variables, obtained from the survey, one value was connected to each farm while at the field level the mean value of four samples were considered for both X and Y variables. Both PLS matrices can be expressed as: Y = TQ’ + F and X = TP’ + E, where matrix T contains X scores, the P matrix contains X loadings, matrix Q contains the Y loadings and F and E matrices are the residuals of the un-explained variation in Y and X tables. The relationship among the Y and X tables was derived through the latent variable T. The latent T variable represents the proportion of the explained interaction variance of the Y matrix by the set of variables from the X matrix. The number of T variables, or principal components (PC1 and PC2) in our figures, that are requested to optimally predict the dimensionality of the Y matrix, was determined by cross-validation procedure^22^.

As the performance of the PLS regression models improves with relevant X-variables that explain the most variation of Y variables, we used the filter method with the variable importance in the projection (VIP) for variable selection^39,40^. This means that after the first model run including all the 34 X-variables, all variables with a VIP less than 1 were eliminated. A second model run with the remaining variables was done. Once the most valid model was reached, we obtained the model fit ability (cumulative R^2^Y, denoted R^2^Y (cum)) and the model predictive ability (cumulative Q^2^Y, denoted Q^2^Y (cum)) for all the dependent variables together and for individual dependent variables. Root mean square relative error (RMSE, %) was also calculated to measure the predictability of the PLS model. As the barley performance was measured with different indicators with different units and scales, the relative error is more meaningful than the absolute error. The RMSRE was calculated as

$${\rm{RMSRE}}=100\times \sqrt{\frac{1}{N}{\sum }_{i=1}^{N}({\frac{yi-\widehat{yi}}{yi})}^{2}}$$

where *yi* is the observed value of the ^i^th measured indicator, $\widehat{yi}$ is the corresponding PLS simulated value and N is the total number of fields.

A total of nineteen sets of X-matrices in relation to the barley performance (Y) were considered in the analyses. Three X-matrices (firstly, all the CF, YOF and OOF (*Model* 1: *n* = 34); secondly only YOF and OOF (*Model* 2: *n* = 22) and thirdly only CF (*Model* 3: *n* = 12)) were analysed and are fully presented as they fitted well with our study objective. The other 16 combinations of X-matrices were analysed to exclude the artefact that might be caused by the unbalanced contribution of different farm types. These included 6 matrices of 12 CF with 12 OF (*Models* 4–9, *n* = 24) and another 10 matrices with combinations of 6 OF from each region (*Model* 10–19, *n* = 12). The PLS analyses were performed with the software SIMCA-P V 13.0 (Umetrics, Umeå, Sweden).

## Results

### Factors influencing crop performance

**Figure 2:**
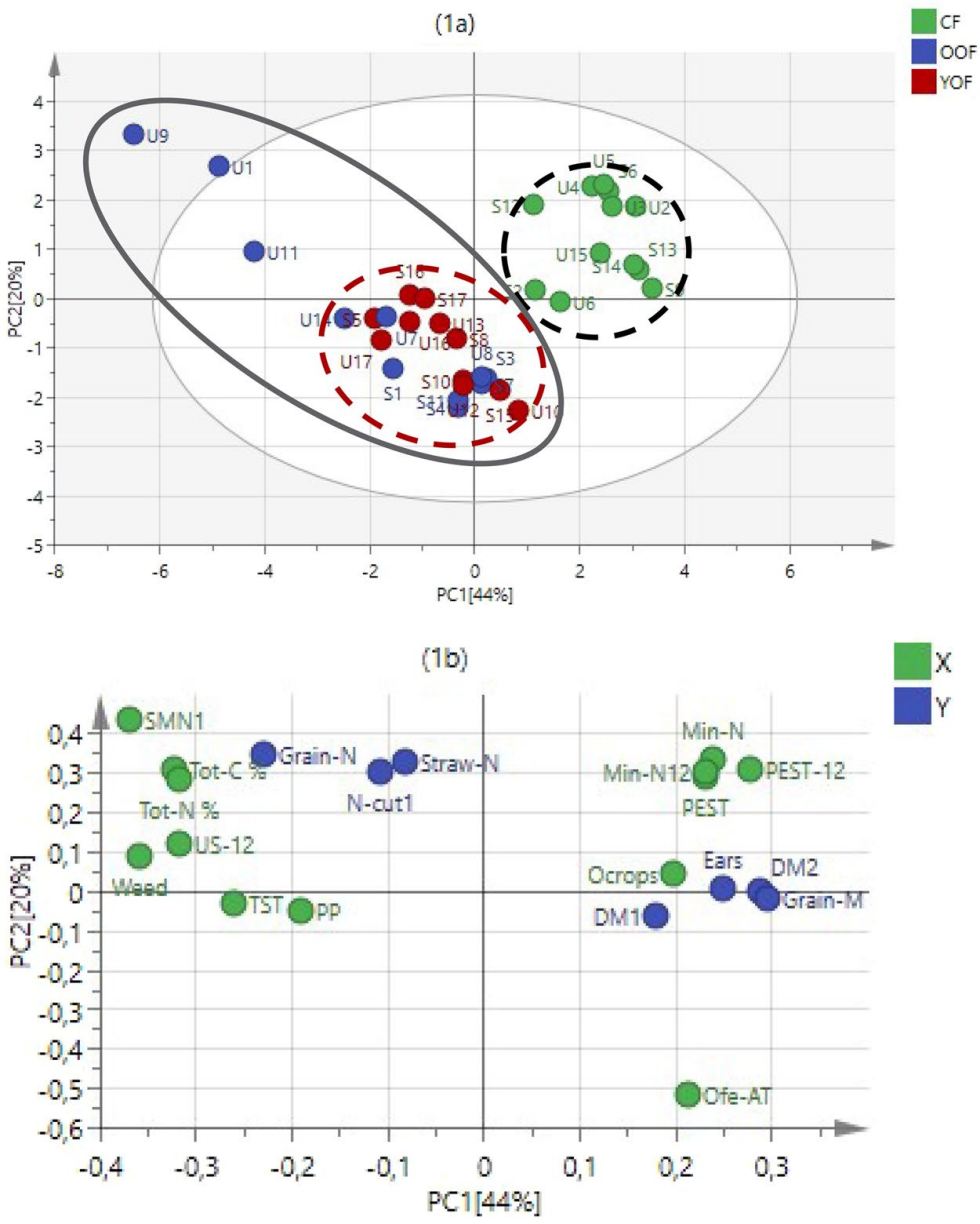
Relationships between management practices, soil characteristics and barley performance indicators (BPIs) for all the farms (n = 34). (**1a**) PLS scores with two main clusters: old OFs with a wide variability in effect of management practices and soil characteristics in a continuous black ellipse together with young OFs in red dashed circle with less variability and all the CFs in blue dashed circle; (**1b**) PLS loading of X (management practices and soil characteristics) and Y variables (BPIs, response variables). Y variables are: dry matter at stem elongation (DM1) and its N concentration (N-Cut 1), total dry matter (DM2), grain dry matter (Grain-M) and its N concentration (Grain-N), N concentration in straw at grain ripening (Straw-N) and the number of ears m^−2^ (Ears). X variables, i.e. management practices retained in the PLS analysis were soil mineral N before fertilization (SMN1), percentage weed cover (weed), total soil C (Tot-C%), barley under-sown with grass/clover in 2012 (US-12), total soil N (Tot-N%), pesticide use in 2012 (PEST-12), time since transition (TST), mineral N use from 2009 (Min-N), pesticide use from 2009 (PEST), application technique of organic fertilizers (Ofe-AT), proportions of other crops (Ocrops) and presence of pasture on the farm (PP) (Table S2a).

**Figure 3:**
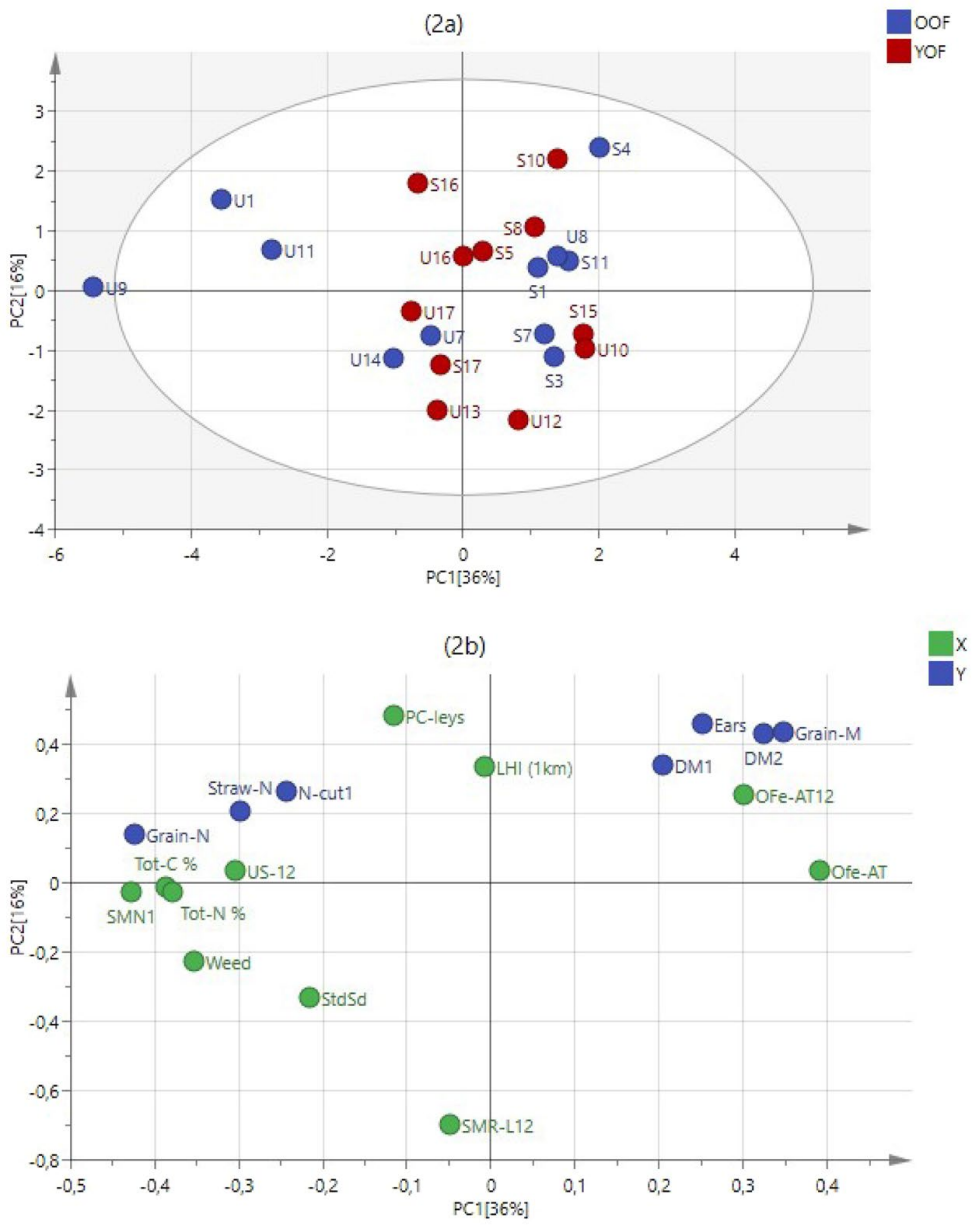
Relationships between management practices, soil characteristics and barley performance indicators for the OF (n = 22). (**a**) PLS with a mixture of old and young OFs with farms from Uppsala (U) and Scania (S); (**b**) PLS loading of X (management practices and soil characteristics) and Y (BPI), response variables. Y variables are: dry matter at stem elongation (DM1) and its N concentration (N-Cut 1), total dry matter (DM2), grain dry matter (Grain-M) and its N concentration (Grain-N), N concentration in straw at grain ripening (Straw-N) and the number of ears m^−2^ (Ears). X variables are; straw and residues left on the field in the year before 2012 (SMR-L12), soil mineral N before fertilization (SMN1), ley as a preceding crop (PC-leys), percentage weed cover (weed), total soil C (Tot-C%), barley under-sown with grass/clover in 2012 (US-12), total soil N (Tot-N%), application technique of organic fertilizers in 2012 (Ofe-AT12), Standardized sowing date (StdSd) and Landscape heterogeneity index 1 km radius (LHI (1 km) (Table S2b).

On conventional farms barley performance was dominated by inputs of mineral fertilisers and pesticides, but on organic farms by a combination of social, ecological and agronomic factors. We found that a combination of seven management practices, three soil parameters and landscape heterogeneity were the major sources of variation explaining barley performance on organic farms (OF), in contrast to conventional farms (CF) where external inputs in the year of study (mineral fertilisers and pesticides) were most important for crop performance (Figs. 2 and 3).

By analysing the OF and CF farms together and separately, we could tease apart the influence of different factors on barley performance in the two farming systems (Tables S2a–c). Among the soil parameters, soil mineral nitrogen content before fertilisation in the year of the study (2012; SMN1), total carbon content (Tot-C%) and total nitrogen content (Tot-N%) were important (VIP1 > 1 in Table S2a,b) when all farms or only the OF were included in the analysis, as were weed cover (Weed) and undersowing of clover and grass in barley (US-12) (Figs. 2 and 3, Table S2a,b). The time since transition to organic farming (TST), the presence of pasture and the proportions of crops other than cereals in the rotation were important for barley performance when all farms were analysed, but these variables ranked among the less important ones when only OF was considered. The analysis of OF alone showed instead that factors such as straw removal prior to barley sowing in the year of performance measurement (SMR-L12), manure application technique in the current year (Ofe-AT12), sowing date (stdSd), ley as preceding crop (PC-leys) and the landscape heterogeneity index (LHI, 1 km) were of high importance. Thus, factors from all classes of variables (Table S1) – landscape, farm management, field management in the present and previous years, and soil factors– were important to explain variation among organic farms. When both farm types were analysed together, historical management practices and their indirect impacts were mostly observed in OF, as shown by the variables such as percentage weed cover (weed), soil mineral nitrogen (SMN1) and total carbon (Tot-C%) that were higher in older farms under organic farming (Fig. 2).

To ensure that the results were not influenced by having twice as many organic as conventional farms in the study, we also analysed an equal number of the two types of farms, with six times random selections of 6 OF and 6 CF in each agro-ecological zone, i.e. in total 24 farms. This did not significantly change the PLS scores indicating the importance of variables in the projections. Using an equal number of conventional and organic farms showed between 14 and 17 factors explaining the variation in barley performance. The 13 variables that were important when all farms were included were retained in all the combinations, but 1 to 4 new variables appeared to be important in one or another combination (Table S2d).

Regardless the types of farms included in the analyses, the models explained more of the variation in OF than in CF. Factors retained as important variables (Table S2a–c) explained 27% (only CF), 38% (combined CF and OF) and 53% (only OF) of the barley performance variation. The corresponding model predictions were 6, 21 and 30%. The average explained variation for twelve randomly chosen OF (n = 12, 6 OF each region) in ten different choices gave a model fit of 48 (SD ± 17)% and a model prediction of 17 (SD ± 7)%. Figure 1 clearly illustrates the higher similarity of the scores among CF compared to OF with a large heterogeneity in scores. None of the soil parameters were important in explaining the variation of barley performance in CF, while soil mineral nitrogen (SMN1), total nitrogen (Tot-N%) and total carbon (Tot-C%) contents explained much of the variation in OF.

### Importance of management history for the analysis of crop performance

Time since transition (TST) to organic farming was key to explaining the variability of barley performance when all farms were compared. When the CFs were excluded, management factors were more important than TST in explaining barley performance. There was more variation among management practices and soil characteristics in Uppsala than in Scania, and this affected barley performance in different ways.

There was a clear separation of young and old organic farms in Uppsala (Fig. 2). The five farms in Uppsala, which had been organic for the longest period, with a time since transition to OF between 17 and 26 years (farms U9, U1, U11, U7 and U14), showed higher scores associated with soil parameters and management practices (Fig. 2). However, Scania farms with the highest number of years since transition to OF (S1: 17 years and S3: 18 years) did not fall into the group of ‘old farms’ in the analysis. When analysing OF alone, the first principal component differentiated between old organic farms in each region while young organic farms did not show the same magnitude of variation as the old ones in terms of management practices and soil characteristics (Figs. 2 and 3).

With different factors influencing the groups of farms, higher dry matter production was observed in CF than in OF, but the grain N concentration was slightly higher on OF. Three old OF in Uppsala (U9, U1 and U11) stand out as having higher N concentrations in the grains (3.7, 2.1 and 2.2%) and less dry biomass at harvest than the rest of the farms (Fig. 3a). Weed cover was negatively related to yield associated variables, i.e. DM1, DM2, grain-M and ears (Fig. 3b). Percentage weed cover was particularly high in some of the old OF with 55, 50 and 12% for U9, U1 and U11, respectively. The techniques used for applying organic fertilizers during the years 2009 to 2012 was also important for barley performance (Ofe-AT and Ofe-AT12 in Fig. 3b). High total soil C and N, and soil mineral N before fertilization were strongly associated with the above three old OF in Uppsala. According to the first principal component (PC1), the OF in Scania tended to have higher biomass production (DM1, DM2 and Grain yields) than the farms in Uppsala, but one OF farm in Uppsala (U8) had a similar performance to the Scania farms (Fig. 3a).

### Explained variation of individual crop performance indicators

Management practices explained more of the variation in grain yield and grain N concentration than that of the other barley performance indicators (BPIs). The explained variation of the seven performance indicators ranged from 19 to 75% (see R^2^Y in Table 3). The amount of dry matter harvested at the beginning of stem elongation (DM1) had the lowest amount of explained variation by the retained factors (with a root mean square relative error (RMSRE) of about 10%) regardless of the group of farms included in the analysis. The variation was best explained for grain yield with an error of around 5% (Table 3). The model prediction ability, obtained by cross-validation, showed a goodness of prediction ranging between 9 and 57% of explained variation of BPIs by important factors (See Q^2^Y in Table 1). For all these individual BPIs, the goodness of prediction on OF only was higher than when all farms were analysed together. The relationships between predicted and observed values of grain yields and N concentrations in grains for all the farms were best with 48 and 59% explained variation, respectively (Fig. S1). Other comparisons of predicted values against observed values are shown in the Supplementary Material (Figs. S2 and S3).

**Table 3:** Model performance indicators (goodness of fit: R^2^Y (0–1), goodness of prediction: Q^2^Y (0–1) and Root Mean Square Relative Error (RMSRE, %) for the analysis including all farms (Model 1, n = 34), OF (Model 2, n = 22) and CF (Model 3, n = 12) in a linear relationship between model predicted and observed values.

Indicators	Model	Grain yield	Grain [N]	DM1	[N] cut 1	DM2	# Ears. m^−2^	Straw [N]
R^2^Y	CF & OF (Model 1)	0.48	0.59	0.19	0.29	0.45	0.34	0.31
	OF (Model 2))	0.75	0.68	0.34	0.31	0.68	0.57	0.39
	CF (Model 3)	0.26	0.53	0.00	0.26	0.05	0.02	0.81
Q^2^Y	CF & OF (Model 1)	0.37	0.25	0.02	0.17	0.35	0.23	0.12
	OF (Model 2)	0.57	0.23	0.18	0.15	0.47	0.35	0.09
	CF (Model 3)	−0.06	0.26	−0.04	0.13	−0.10	−0.10	0.56
RMSRE (%)	CF & OF (Model 1)	5.8	3.0	9.8	5.6	5.8	5.4	5.6
	OF (Model 2)	4.8	3.2	9.5	6.6	5.1	4.6	5.8
	CF (Model 3)	19	9	105	28	21	30	16

Barley performance indicators were grain yield, grain N concentration [N], dry matter at the first and second cut (DM1; DM2) and the number of ears m^−2^. The indicator Q2 was obtained after cross-validation of the model.

## Discussion

We found that more management practices were important for explaining crop performance on organic compared to conventional farms, suggesting that OFs are more complex systems. Furthermore, we found that soil parameters together with weeds were the main factors causing the differences between CF and OF. Our results showed a larger variability of barley performance in OF than in CF, especially among old OF (Figs. 1 and 2). Crop performance in organic farming thus appeared to depend on more factors than in conventional farming. Furthermore, the variation in crop performance among conventional farms was much smaller than among organic farms. Our results agree with previous findings that there can be large heterogeneity of management practices within the same farming systems^23,41^, meaning that studies need to be carried out within as well as between farming systems.

By expanding datasets to two agro-ecological zones and including soil parameters in the PLS analysis, it was clear that the heterogeneity of management practices affecting crop performance indicators became larger. Some of the OFs, such as U9 and U1, ended up outside the 95% interval of confidence of the model (Fig. 2), which in the analysis of factorial experiments would classify them as outliers. Such extreme observations are interesting because extreme events are part of reality and cannot be ignored in the analysis of complex systems^24^. On these farms, we observed three common characteristics. Firstly weed cover percentage was approximately four times higher than the average (U1 with 55% and U11 with 50% weed cover in relation to 12% in average). Secondly, the three farms had higher levels of total soil C (around 12, 9 and 5% for U1, U9 and U11, respectively) and thirdly they had higher soil mineral N before fertilizer application than the other farms. Both the high abundance of weeds and the high grain N concentrations on these farms can be explained by the high soil organic matter and high N mineralization rates from the soil. Contrasting farms such as farm U9 and U8, which differed in, e.g. application of organic fertilizer (OFe-AT and OFe-AT12), might aid understanding of which variables are causing low or high biomass production, and thus provide design criteria for further experiments and recommendations for improving crop performance.

### Extending the temporal and spatial boundaries is important for complex social-ecological systems

Factors influencing yield and other crop performance indicators were shown to be related to different periods in the farm history (e.g. time since transition (TST), management practices the year of growing barley, or the years before) as well as factors including landscape heterogeneity, crop rotation, etc. These findings show how complex sets of farm variables can influence indicators of crop performance. The importance of both temporal and spatial factors in affecting barley performance strongly suggests a need to utilize a systems perspective in order to understand crop performance in agro-ecosystems. Short-term versus long-term solutions used by conventional and organic farmers need to be integrated in the understanding of the farming systems^42^ to address the complex interaction networks of ecological factors and human decisions^29^. Our analysis of management practices and soils on organic farms revealed how diverse the farms can be within one type of farming and one agro-ecological zone (Fig. 3). Conventional assessment methods with experiments, contrasting a limited number of factors (e.g. management practices, soil conditions), would not have revealed this and thus such methods would have limited the understanding of the many interacting factors affecting yields. Our results show that on farm data collection integrates a diversity of farm practices and consequences over time, which are the combined effect of social and ecological conditions on a farm. In that way, on-farm studies in combination with PLS can be regarded as an effective way to assess the effects of management practices on crop performances but also on other ecosystem services including supporting ecosystem services not examined here, such as pollination or biological control^37^.

### Performance indicators with direct economic benefits were well predicted by the studied factors

The variables of high importance (VIP, Table S2a–c) as classified by PLS include the choices made by farmers, which influence plant performance indicators and have economic significance. Grain yield was the best predicted performance variable with 48% explained variation for all farms and 75% for OF only (Table 3). The better prediction of grain yields for OF could be explained by their larger heterogeneity in terms of the choices of management practices, in comparison to CF that were more homogenous in management practices and relying primarily on mineral fertilizers and pesticides to support the grain production. Consequently, it can be seen in Fig. 2 that some OFs can produce yields (Grain-M) which are almost equal to conventional farm. The two OF, U8 and S10, showed yields of 5.1 and 6.5 tons per hectare, larger than the CF average of 4.9 tons per hectare in the study year 2012), but OFs can also perform quite poorly. PLS can be used as a method to identify different strategies and goals among farmers and to evaluate other ecosystem services delivered by agroecosystems. PLS can also be used to identify the most relevant factors to study in future experiments, thus improving their design and cost effectiveness. Studies using the PLS method including both crop performance and other ecosystem services as response variables are thus likely to be important tools for identifying factors influencing crop performance in future. It should also be possible to use some indirect ecosystem services, such as biological control^43^, pollination or earthworm activity, as explanatory variables in a PLS, provided that these can be adequately measured.

### The PLS method has the potential to integrate scientific disciplines in the analysis of socio-ecological systems

In this study, we examined a set of factors for crop production that traditionally are of concern in different scientific disciplines. Soil C and N and soil management practices and their effect on the crop yield is normally studied by soil scientists^44^. Ecologists are often interested in how the landscape (here heterogeneity index, LHI) affects the ecosystem services and functions^43,45,46^, while plant scientists and agronomists are mainly interested in, for example, weeds, crop rotations and crop yields in agro-ecosystems^47^. Social scientists are interested in the choices and views of farmers on for example biodiversity and climate change adaptation^48^. PLS is thus a bridging method that provides a mechanism to foster a multidisciplinary approach to the analysis of complex agro-ecosystems. The principle of multidisciplinarity using PLS was also argued as a good way to tackle the understanding of the complexity of financial systems^24^. Factors embedded in the interactions of genotype, management and environment, which are usually regarded as determinants of crop yields^49^, could therefore be integrated with regard to farmer decisions of social and ecological origin. At the genotype level, choice of cultivars depends on the purpose (feed, or malting in case of barley), the market demand and how farmers value production for home consumption. The choice of management practices is a function of the socio-economic conditions including the technological and financial possibilities. The farmed environment goes beyond the weather and soils that are normally considered in process-based modelling and multi-environment trials. Our study indicates that social dimensions (e.g. learning and decisional ability – reflected in the factors time since transition and choice of management practices) interact with the environment to affect the outcomes in managed ecosystems.

Despite the advantage of the PLS regression method in accounting for multi-dimensional aspects of the complexity of agroecosystems, one weakness is worth mentioning, namely that the causality between the factors and responses are not straightforward. PLS regression coefficients refer to created reduced dimensions from the data^40^. An alternative way to establish causal relationships among networks of different groups of variables^50^ would have been PLS Path Analysis (PLS-PA) or Structural Equation Modelling (SEM), which are rather different methods^51^ and also used in other disciplines dealing with complexity such as psychology^52^, marketing^53^, information systems^54^ and ecology^50^. In this study, PLS-PA and SEM could not be applied because they require a higher ratio between the number of objects (sample size: in our case the number of farms) and the number of variables included in the analysis, which has been estimated to be at least 5 and preferably 10^27^. In this case, we would have needed a minimum of 170 farms in the study for the 34 variables used, which would entail substantial resources (human and financial). The models developed with PLS regressions, however, are satisfactory considering the prediction levels of barley performance indicators (Figs. S1, S2 and S3; Table 3).

In conclusion, crop performance in organic farming systems was explained by a diverse set of factors; previous and current choices of management as well as landscape and soil variables. Extending the temporal and spatial boundaries is important for complex social-ecological factors which influence crop performance. Comparing organic and conventional farms, the main difference was in fertilizer and pesticide use, which was expected. In contrast, the conventional farming model explained little variation in crop performance among the farms. Finally, we have demonstrated how complex agricultural production systems can be analysed using on-farm data collection, integrating a range of disciplines and using PLS regressions. While the analysis method requires minimal data it still appears to be robust. It is exploratory but it also provides a predictive dimension, which evaluates the model usefulness for each individual measure of crop performance or other ecosystem services of interest, and it can thus inform the planning of new experimental work by improving design and cost effectiveness.

## Supplementary information

Supplementary information.
