# Supplementary material for: Socio-ecological factors determine crop performance in agricultural systems
Source: Sci Rep. 2020 Mar 6;10:4232. doi: 10.1038/s41598-020-60927-1 (PMC7060324; doi:10.1038/s41598-020-60927-1)
Supplement: Supplementary file 1 — Supplementary information. [file 41598_2020_60927_MOESM1_ESM.pdf]

Supplementary material

Socio-ecological factors determine crop performance in agricultural systems

Libère Nkurunziza[1,*], Christine A Watson[1,2], Ingrid Öborn[1], Henrik G. Smith[3], Göran Bergkvist[1] and Jan Bengtsson[4]

[1]*Department of Crop Production Ecology, Swedish University of Agricultural Sciences (SLU), P O Box 7043, SE 75007 Uppsala, Sweden*

[2]*Scotland's Rural College (SRUC), Craibstone Estate, Aberdeen AB21 9YA, Scotland*

[3]*Centre for Environmental and Climate Research & Department of Biology, Lund University, SE 223 62 Lund, Sweden*

[4]*Department of Ecology, SLU, P O Box 7044, SE 75007 Uppsala, Sweden*

**Corresponding author: E-mail: libere.nkurunziza@slu.se*

Table S1: Selected questions from the questionnaire to farmers on the management practices (MP), examples of answers registered and corresponding variables used in the PLS analysis

Questions asked	Answers registered	Corresponding variable (V) in Table 2
**Farm level description and MP**		
1. What is the size of your farm?	Number of hectares	V2: Farm size
2. Since when did you convert from conventional to organic farming?	The year	V1: Time since transition
3. What is the crop distribution (ha)	Number of hectares of rotational leys, grains, oil seeds, pasture, permanent pasture and other	V4-7: Proportions of the crops
5. How much organic fertilizer do you use on the farm?	The area (ha), type of organic fertilizer, amounts	V8: area and V9: amounts
6. How many animals do you have on the farm?	Number of cattle, pigs, sheep, poultry, horse	V10: Livestock density index
8. How are straw and other crop residues managed on the farm?	Left on the farm, stubble cultivation, ploughed, removed or differs from time to time	V11: Straw and residues management
**Field level MP (2009 -2011)**		
9. How much mineral fertilizers used Mineral fertilizer, field 2009	Type of mineral fertilizer and amounts	V13-14: Mineral fertilizers (NPK)
10. Were pesticides used on the field in 2009?	Herbicides, insecticides, fungicides and organic pesticides	V16: Pesticide application
11. Were organic fertilizers spread on the field of 2011?	Autumn 2010, spring 2011, other time, if yes what type and amount (tons/ha)	V13: Organic fertilizers application technique
12. How often do you spread organic fertilizer on this field?	Yearly, other periodicity	
13. What distribution technique was used?	Broadcasting, row spraying, other methods	
14. Were the fertilizers mulched after application?	Yes or No	
15. Were any mineral fertilizers used on this field?	Amount in kg of N, P, K	V14: Mineral N
16. How was straw and other crop residues managed in this field 2010?	Left on the field, ploughed, removed or other	V17: Straw and residues management
17. How was straw and other crop residues managed in this field 2011?	Left on the field, ploughed, removed or other	
18. Were pesticides used in 2011?	Herbicides, insecticides, fungicides	V16: Pesticide application
**Field level MP in 2012**		
19. Was organic fertilizer spread in the field of 2012?	Autumn 2011, spring 2012, other time, type and the amount (tons)	V18: Amount of organic fertilizers
20. What distribution technique was used for organic fertilizers in 2012?	Broadcasting, row spraying or other method	V19: Ofe application technique
21. Were the fertilizers mulched after application in 2012?	Yes or No	
22. Were any mineral fertilizers used on this field in 2012?	Amount of N, P, K (kg) and the application date	V20: Mineral N
23. How were straw and residues managed in the same field in 2012?	Left on the field, ploughed, removed or other method	V21: Straw and residues left on the field
24. What was the sowing date in 2012	Date	V22: Sowing date
25. What was the seed rate in 2012?	kg/ha	V23: Seed rate sown
26. Was any crop undersown in the barley 2012?	Species, proportion	V28: Undersown barley
27. What was the preceding crop in 2012?	Ley, grain, oilseed, sugar beet, permanent pasture, other crop	V24-26: preceding crops
28. Were pesticides applied in 2012?	Yes or No for herbicides, insecticides, fungicides	V27: Use of pesticide

Table S2a: Variable importance in the projections (VIP) for barley performance indicators with all farms (n=34). VIP1 and VIP2 indicate VIP before and after elimination of variables of less importance, i.e. variables with*VIP1<1*. SE1 and SE2 are standard error after model cross validation before and after elimination of variables of less importance. “Rank” is the final variable ranks according to the VIP2 values.

**Retained variables (VIP >1) at the first model run**						
	**Symbol**	**VIP1**	**SE1**	**VIP2**	**SE2**	**Rank**
Soil mineral nitrogen before fertilisation 2012	SMN1	1.95	2.73	1.34	1.51	1
Percentage weed cover	Weed	1.90	1.06	1.08	0.33	5
Total soil carbon	Tot-C %	1.71	2.31	1.10	0.98	3
Barley under-sown with grass/clover in 2012	US-12	1.69	0.82	0.97	0.76	8
Total soil nitrogen	Tot-N %	1.67	2.15	1.06	0.88	6
Pesticides used in 2012	PEST-12	1.47	1.81	1.09	0.92	4
Time since transition to OF	TST	1.37	1.16	0.80	0.53	11
Mineral N use from 2009	Min-N	1.26	1.54	1.03	0.73	7
Mineral N use in 2012	Min-N12	1.22	1.64	0.97	0.71	9
Pesticides used from 2009	PEST	1.22	1.35	0.95	0.55	10
Application technique of organic fertilizers	Ofe-AT	1.13	1.27	1.14	0.52	2
Proportions of other crops	Ocrops	1.04	0.63	0.61	0.46	12
Presence of pasture on the farm	PP[1]	1.01	0.54	0.60	0.36	13
**Eliminated variables (VIP<1) after first model run**						
Standardized sowing date	StdSd	0.91	1.06			
Straw and residue management (2009-11)	STR-M	0.84	1.50			
Standardized soil clay content	Clay sdrt	0.81	1.84			
Proportion of rotational leys on arable land	Leys	0.80	1.16			
Mineral PK applied (2009-11)	Min-PK	0.78	1.89			
pH	pH	0.70	1.24			
Application technique of organic fertilizers in 2012	OFe-AT12	0.69	0.94			
Proportion of cereals on arable land	Cereal	0.63	1.07			
Straw and residues left on the field the year before 2012	SMR-L12	0.47	2.78			
Livestock density index	LDI	0.47	1.17			
Ley as preceding crop	PC-leys	0.44	1.33			
Nitrogen amount from organic fertilizers 2012	Org-N12	0.43	1.35			
Amount of organic fertilizer per hectare (2009-11)	Ofe/ha	0.32	1.26			
Cereal as preceding crop	PC-Cereal	0.32	1.27			
Farm size	Size (ha)	0.28	0.93			
Landscape heterogeneity index 1km radius	LHI (1km)	0.22	0.62			
Seed rate	Seed rate	0.18	0.67			
Straw and residue management at the whole farm	SRM	0.18	0.68			
Frequency of organic fertilizer	Freq-Ofe	0.11	1.19			
Area with organic fertilizer on farm	Ofe-area[2]	0.09	0.89			
Other crop than cereals as preceding crop	PC-other	0.04	1.01			

Table S2b: Variable importance in the projections (VIP) for barley performance indicators with only organic farms (n=22). VPI1 and VIP2 indicate VIP before and after elimination of variables of less importance, i.e. variables with*VIP1<1*. SE1 and SE2 are standard error after model cross validation before and after elimination of variables of less importance. “Rank” is the final variable ranks according to the VIP2 values.

**Retained variables with VIP >1 at the first model run**						
**Variables**	**Symbol**	**VIP1**	**SE1**	**VIP2**	**SE2**	**Rank**
Straw and residues left on the field the year before 2012	SMR-L12	1.89	1.13	1.37	1.09	1
Soil mineral nitrogen before fertilisation 2012	SMN1	1.58	1.80	1.14	1.48	2
Leys as preceding crop	PC-leys	1.44	0.72	1.06	0.76	3
Application technique of organic fertilizers	Ofe-AT	1.44	0.51	1.04	0.11	4
Total soil carbon	Tot-C %	1.40	1.09	1.04	0.72	5
Percentage weed cover	Weed	1.37	1.30	0.98	0.78	7
Total soil nitrogen	Tot-N %	1.37	0.94	1.01	0.59	6
Application technique of organic fertilizers in 2012	OFe-AT12	1.22	1.30	0.87	0.74	8
Barley under-sown with grass/clover in 2012	US-12	1.13	0.95	0.83	0.39	9
Standardized sowing date	StdSd	1.09	1.06	0.80	0.94	10
Landscape heterogeneity index 1km radius	LHI (1km)	1.02	1.53	0.68	1.34	11
**Eliminated variables (VIP<1) after the first model run**						
Cereal as preceding crop	PC-Cereal	0.94	0.95			
Standardized soil clay content	Clay sdrt	0.88	0.91			
Other crop than cereals and ley as preceding crop	PC-other	0.87	1.46			
Time since transition to OF	TST	0.87	1.30			
Proportion of rotational leys in the farm	Leys	0.86	0.83			
Livestock density index	LDI	0.77	0.77			
Proportion of cereals on arable land	Cereal	0.71	0.87			
Presence of pasture on the farm	PP	0.65	0.98			
pH	pH	0.63	0.78			
Amount of organic fertilizer per hectare (2009-11)	Ofe/ha	0.62	1.25			
Proportion of other crops than cereals	Ocrops	0.56	0.96			
Nitrogen amount from organic fertilizers 2012	Org-N12	0.53	0.88			
Frequency of organic fertilizer	Freq-Ofe	0.52	1.15			
Straw and residue management (2009-11)	STR-M	0.50	0.93			
Straw and residue management at the whole farm	SRM	0.49	0.91			
Farm size	Size (ha)	0.49	1.17			
Seed rate of barley	Seed rate	0.42	0.76			
Area with organic fertilizer on farm	Ofe-area	0.32	0.95			

Table S2c: Variable importance in the projections (VIP) for barley performance indicators with only conventional farms (n=12). VIP1 and VIP2 indicate VIP before and after elimination of variables of less importance, i.e. variables with*VIP1<1*. SE1 and SE2 are standard error after model cross validation before and after elimination of variables of less importance. "Rank" is the final variable ranks according to the VIP2 values.

**Retained variables (VIP >1) at the first model run**	**Symbol**	**VIP1**	**SE1**	**VIP2**	**SE2**	**Rank**
Frequency of organic fertilizer	Freq-Ofe	1.67	1.89	1.20	1.23	2
Cereal as preceding crop	PC-Cereal	1.63	1.01	1.20	0.84	1
Mineral PK applied (2009-11)	Min-PK	1.56	2.81	1.13	1.56	3
Livestock density index	LDI	1.48	2.18	1.13	1.73	4
Proportion of cereals on arable land	Cereal	1.47	0.90	1.08	0.77	5
Application technique of organic fertilizers	Ofe-AT	1.34	0.85	0.99	0.62	6
Landscape heterogeneity index 1km radius	LHI (1km)	1.28	1.31	0.97	1.22	7
Application technique of organic fertilizers in 2012	OFe-AT12	1.25	0.86	0.91	0.63	10
Farm size	Size (ha)	1.25	2.30	0.96	1.76	8
Seed rate	Seed rate	1.21	3.57	0.95	2.44	9
Proportion of rotational leys on arable land	Leys	1.10	1.44	0.84	1.23	11
Nitrogen amount from organic fertilizers 2012	Org-N12	1.09	2.21	0.76	2.00	12
Area with organic fertilizer on farm	Ofe-area	1.06	1.86	0.75	1.42	13
**Eliminated variables (VIP<1) after the first model run**						
Straw and residue management (2009-11)	STR-M	0.92	2.93			
Amount of organic fertilizer per hectare (2009-11)	Ofe/ha	0.78	1.03			
Total soil carbon	Tot-C %	0.76	1.81			
Straw and residues left on the field the year before 2012	SMR-L12	0.76	1.52			
Total soil nitrogen	Tot-N %	0.75	1.79			
Standardized soil clay content	Clay sdrt	0.68	1.88			
Soil mineral nitrogen before fertilisation 2012	SMN1	0.61	1.42			
Other crop than cereals as preceding crop	PC-other	0.60	1.38			
pH	pH	0.53	1.30			
Mineral N use from 2009	Min-N	0.44	1.71			
Percentage weed cover	Weed	0.44	1.68			
Standardized sowing date	StdSd	0.34	0.47			
Straw and residue management at the whole farm	SRM	0.16	1.47			
Proportions of other crops	Ocrops	0.13	0.78			
Mineral N use in 2012	Min-N12	0.11	0.82			
Presence of pasture on the farm	PP	0.08	1.80			

Table S2d: List of variables of importance in the projections (VIP) for barley performance indicators with different combinations of 12 OF and 12 CF (n=24). Variables 1-13 were retained in the analysis of all the farms (n=34). Variables in *Italic* (nr 14-20) indicate important variables retained in at least one of six PLS analyses with equal number of OF and CF (n=24). Variables nr 21-34 were eliminated in all the combinations.

	**Variables retained at least once (VIP >1) at the first model run**	**Symbol**
1	Soil mineral nitrogen before fertilisation 2012	SMN1
2	Percentage weed cover	Weed
3	Total soil carbon	Tot-C %
4	Barley under-sown with grass/clover in 2012	US-12
5	Total soil nitrogen	Tot-N %
6	Pesticides used in 2012	PEST-12
7	Time since transition to OF	TST
8	Mineral N use from 2009	Min-N
9	Mineral N use in 2012	Min-N12
10	Pesticides used from 2009	PEST
11	Application technique of organic fertilizers	Ofe-AT
12	Proportions of other crops	Ocrops
13	Presence of pasture on the farm	PP[1]
14	*Straw and residue management (2009-11)*	*STR-M*
15	*Standardized soil clay content*	*Clay sdrt*
16	*Mineral PK applied (2009-11)*	*Min-PK*
17	*Application technique of organic fertilizers in 2012*	*OFe-AT12*
18	*Straw and residues left on the field the year before 2012*	*SMR-L12*
19	*Seed rate*	*Seed rate*
20	*Frequency of organic fertilizer*	*Freq-Ofe*
	**Eliminated variables in all combinations (VIP<1) at the first model run**	
21	Standardized sowing date	StdSd
22	Proportion of rotational ley on arable land	Ley
23	pH	pH
24	Proportion of cereals on arable land	Cereal
25	Livestock density index	LDI
26	Ley as preceding crop	PC-leys
27	Nitrogen amount from organic fertilizers 2012	Org-N12
28	Amount of organic fertilizer per hectare (2009-11)	Ofe/ha
29	Cereal as preceding crop	PC-Cereal
30	Farm size	Size (ha)
31	Landscape heterogeneity index 1km radius	LHI (1km)
32	Straw and residue management at the whole farm	SRM
33	Area with organic fertilizer on farm	Ofe-area[2]
34	Other crop than cereals as preceding crop	PC-other

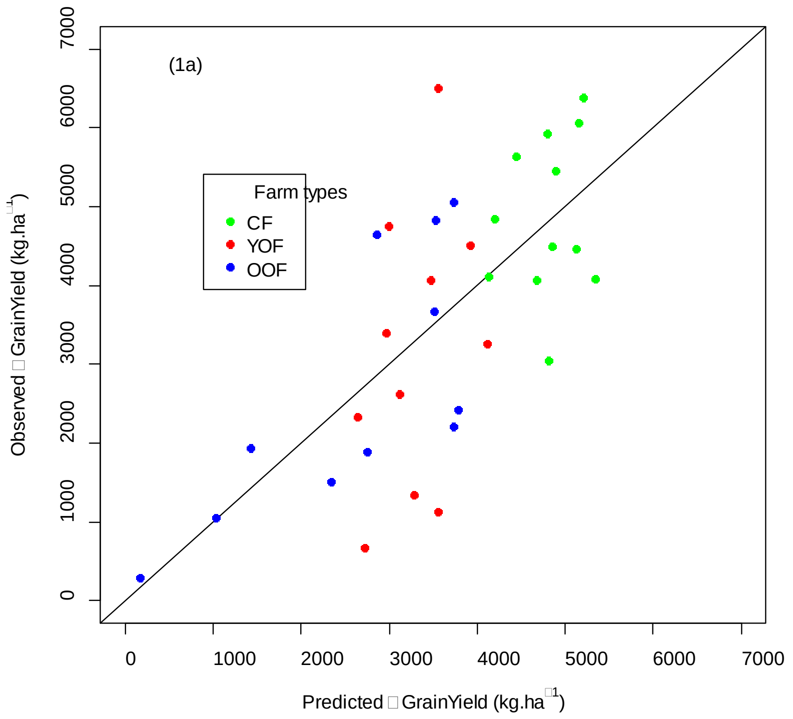

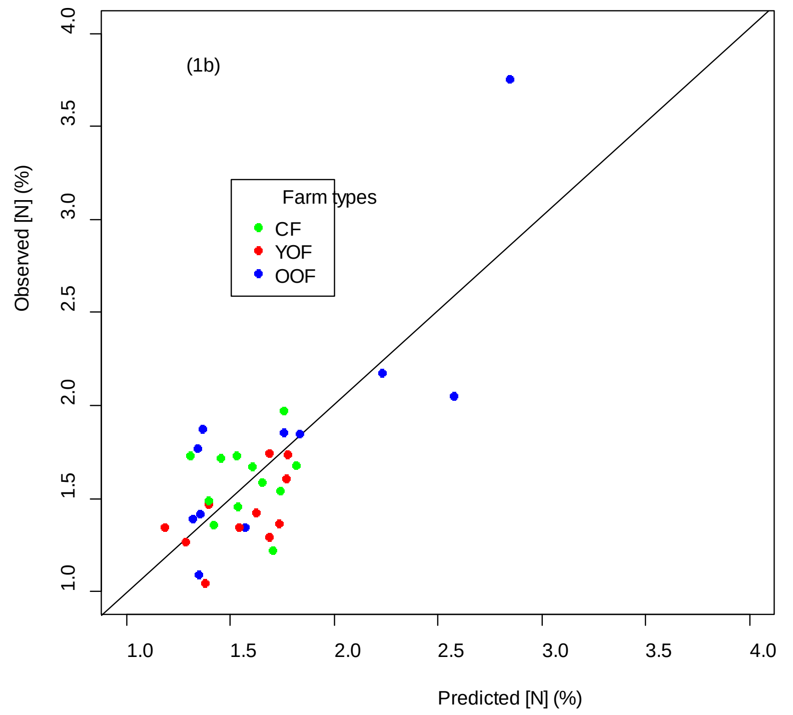

Figure S1: Comparisons between observed and predicted dry matter (DM) of barley 1a) grain biomasses and 1b) nitrogen concentration in barley grains at BBCH 87according the PLS model 1 (n=34). Farm types are represented with different colours.

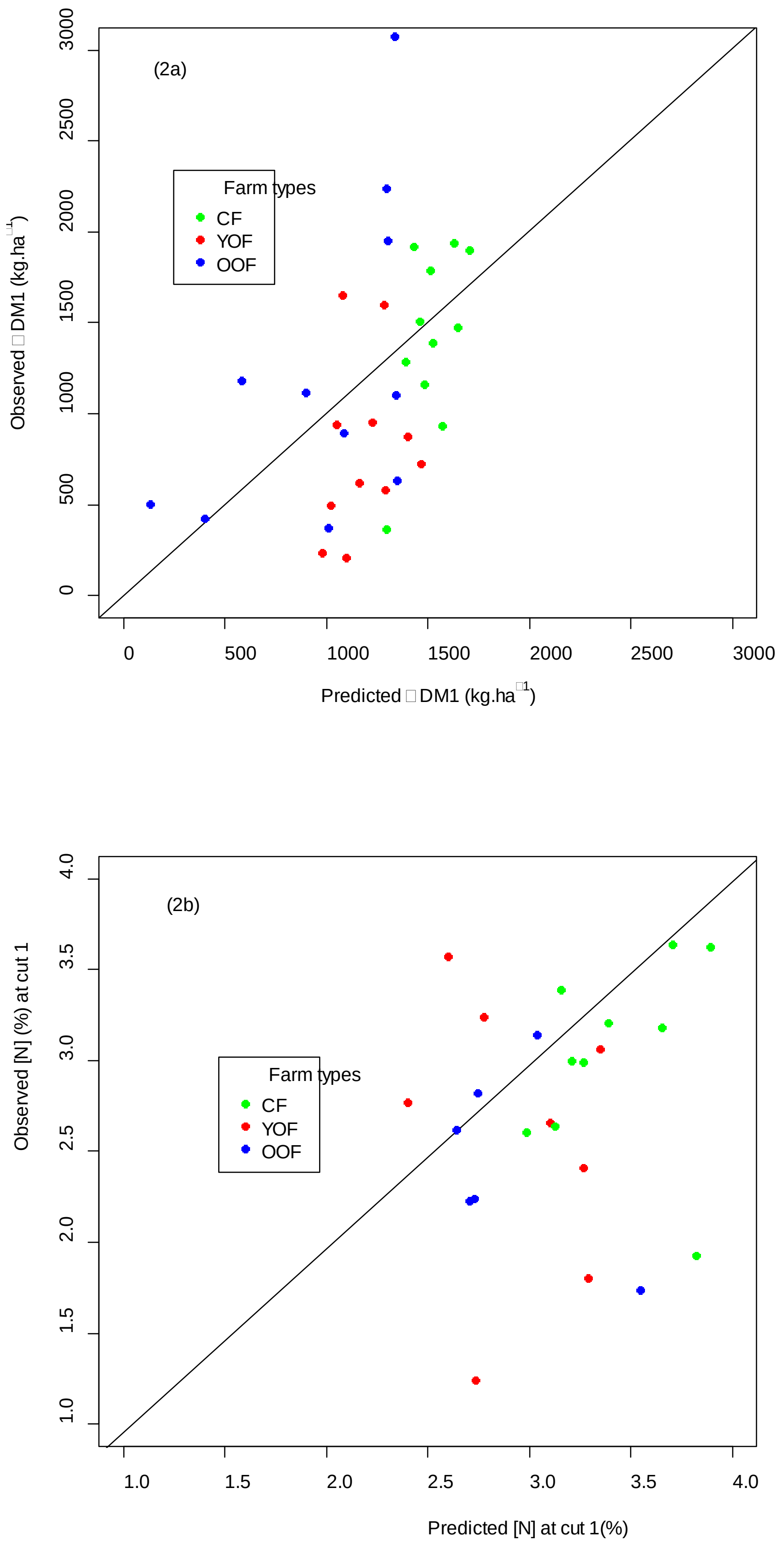

Figure S2: Comparisons between observed and predicted dry matter (DM) of barley biomass at BBCH 31 (2a) and at BBCH 87 (2b) according the PLS model 1 (n=34). Farm types are represented with different colours.

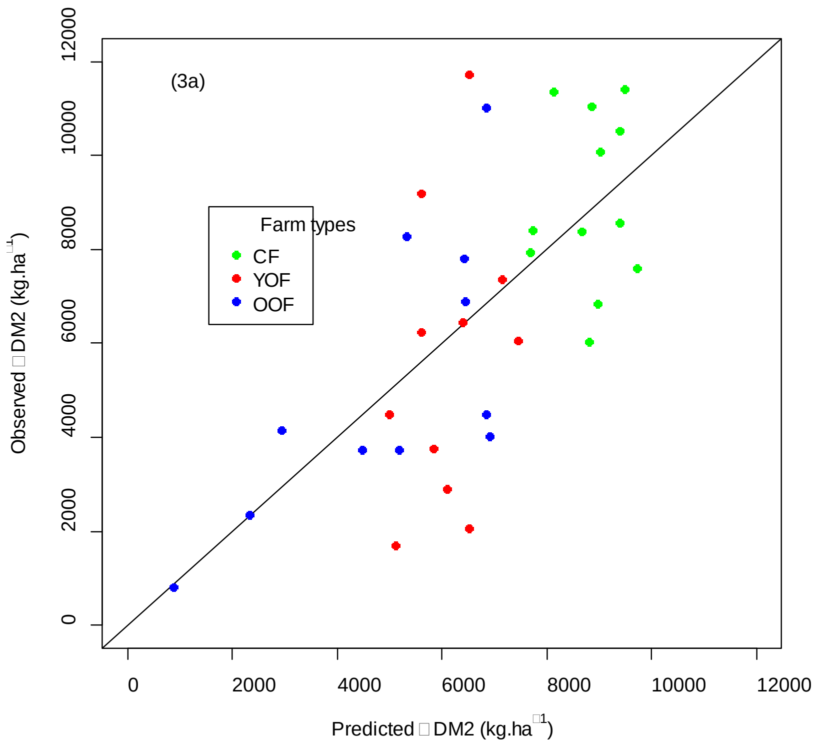

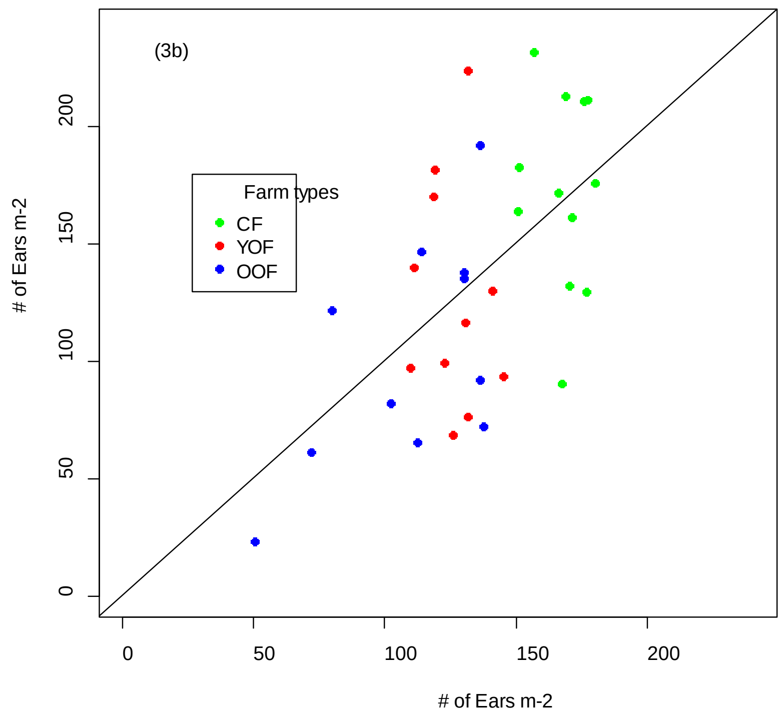

Figure S3: Comparisons between observed and predicted nitrogen concentration [N] of barley biomasses at BBCH 31 (3a) and number of ears m^{-2} at harvest (BBCH 87) (3b) according the PLS model 1 (n=34). Farm types are represented with different colours.
